# AFFECTIONATE TOUCH AND RELATIONAL, MENTAL, AND PHYSICAL WELL-BEING IN OLDER COUPLES: A NATIONAL LONGITUDINAL STUDY

**DOI:** 10.1093/geroni/igz038.2917

**Published:** 2019-11-08

**Authors:** Ruixue Zhaoyang, Lynn M Martire

**Affiliations:** 1 Center for Healthy Aging, The Pennsylvania State University, University Park, Pennsylvania, United States; 2 Human Development and Family Studies, The Pennsylvania State University, University Park, Pennsylvania, United States

## Abstract

Recent theories suggest that non-sexual physical contact with close others plays a key role in promoting health and well-being in adulthood. However, the impact of non-sexual physical contact in later life, especially the affectionate touch between romantic partners, has been largely unexplored. Using two waves of dyadic data (N=953 couples, Mage=71 years) from National Social Life, Health, and Aging Project (NSHAP), we examined whether shared affectionate touch between spouses prospectively predicted both partners’ relational, mental and physical well-being five years later, independent of sex activity. Dyadic analyses results indicated that frequency of shared affectionate touch with the partner predicted increases in spouses’ own relationship satisfaction, life satisfaction and mental health, but not in physical health, over five years. No interpersonal (i.e., partner) effect of shared affectionate touch was found. Findings underscore the unique role of non-sexual physical contact between spouses in promoting relational and mental well-being for older couples.

